# Utilising daily diaries to examine oral health experiences associated with dentine hypersensitivity

**DOI:** 10.1186/s12903-016-0286-9

**Published:** 2016-09-15

**Authors:** Jenny M. Porritt, Farzana Sufi, Sarah R. Baker

**Affiliations:** 1 Department of Psychology, Sociology & Politics, Sheffield Hallam University, Collegiate Crescent, Sheffield, S10 2BQ UK; 2 Clinical Research (Oral Care), GlaxoSmithKline Consumer Healthcare, St Georges Avenue, Weybridge, Surrey KT13 0DE UK; 3 School of Clinical Dentistry, University of Sheffield, 19 Claremont Crescent, Sheffield, S10 2TA UK

**Keywords:** Pain, Dentine hypersensitivity, Coping, Diary, Oral impacts

## Abstract

**Background:**

The current investigation examined the determinants of oral health experiences associated with dentine hypersensitivity using prospective diary methodology.

**Methods:**

Staff and students from a large UK university who had self-diagnosed dentine hypersensitivity completed an online daily diary and text survey for 2 weeks recording their mood, oral health-related coping behaviours, coping and pain appraisals, pain experiences and functional limitations. Cross sectional and lagged path analyses were employed to examine relationships.

**Results:**

One hundred one participants took part in the diary study. Participants had a mean age of 26.3 years (range = 18–63) and most were female (*N* = 69). Individuals who used more oral health-related coping behaviours predicted and experienced greater levels of pain on subsequent days. Negative mood also predicted worse pain outcomes. The daily diary method provided a useful avenue for investigating variations in oral health experiences and relationships between variables that can fluctuate daily.

**Conclusions:**

Psychological variables such as coping and mood play an important role in the pain experiences of people with dentine hypersensitivity. The study highlights the benefits of using prospective methods to elucidate the experiences of people with oral conditions.

## Background

Dentine hypersensitivity affects approximately half of the population and the pain associated with this condition can limit oral functions such as eating and tooth brushing, which negatively affect individual oral health related quality of life [1–5]. Previous research has revealed that key psychological factors can impact on the quality of life of people with dentine hypersensitivity [6], however, the symptoms of this condition are transient and fluctuate frequently. Moreover, even the self-management of dentine hypersensitivity can vary frequently as people may only use products occasionally. For this reason, study designs that enquire about dentine hypersensitivity need to allow adequate investigation of the everyday experience of the condition and factors that may influence this experience.

Coping may play an important role in an individual’s dynamic response to health conditions [7, 8] People may not have consistent styles of coping (e.g. traits) but appear to respond differently in response to specific situations, such as pain [9, 10]. People with dentine hypersensitivity use a number of dentine hypersensitivity-related coping behaviours, which are problem focused (to prevent pain sensations from occurring) such as warming cold food/drinks up before consuming them and the use of toothpaste specially formulated for sensitive teeth [4, 11]. However, there is little research examining how these adaptations are associated with health outcomes in this group of the population. Understanding how well people feel these behaviours help them cope with this oral health condition on a daily basis (e.g. perceived coping efficacy) is also extremely important because positive appraisals of coping are associated with less frequent subsequent pain [12, 13]. Such research would require frequent assessments given the specificity of stimuli and responses.

Another possible predictor of daily pain experience is mood [14, 15]. There is convincing evidence that individual mood and pain experience are intrinsically linked. Low mood both predicts future pain experience [16–18] and may be a consequence of prior pain experience [19]. Clearly this is relevant to a condition with recurring symptoms but to date no research has investigated the relationship between mood, oral health-related coping behaviours and the oral health experiences of dentine hypersensitivity, or indeed any other acutely-experienced oral health condition. Research of this nature, which can expose both within and between-person variation in the oral health experiences related to dentine hypersensitivity, could help clinicians and patients develop a better understanding of the impact and effective management of the condition.

Therefore, to deepen our understanding of how pain experiences are associated with a particular health condition it has been proposed that studies should be prospective and provide more reliable data on how psychological variables and health outcomes may be inter-related and the day-to-day impacts of health conditions [14, 20–22]. Clearly such research requires frequent data collection. Daily diaries offer a unique opportunity to capture day-to-day experiences as they occur thus overcoming the recall bias arising when data are collected retrospectively [14]. Therefore, the current investigation had two aims; firstly, to identify the determinants of the daily experiences of oral health in people with dentine hypersensitivity and secondly, to explore how daily diaries can be used to examine the daily impacts caused by this oral condition. The specific research questions were as follows:

- Do oral health-related coping, perceived coping efficacy and pain predictions successfully predict following day health outcomes in individuals with dentine hypersensitivity?
- Are daily mood and oral health behaviours associated with dentine hypersensitivity-related pain experience?
- What are participants’ experiences of using daily diary methodology to examine dentine hypersensitivity?

## Method

This prospective daily diary study formed part of a larger research project and longitudinal data obtained from a retrospective questionnaire-based study, which examined how key clinical and psychological factors (e.g. illness beliefs) influence the oral health-related and health-related quality of life outcomes in individuals with dentine hypersensitivity, have been published elsewhere [6].

An advertisement for the study was placed on the intranet page of a large UK University available for staff and students to view. Individuals interested in participating in the study completed a pre-study screening questionnaire and a strict exclusion criteria based on Holland et al.’s guidelines [23] were employed. Only those individuals who experienced dentine hypersensitivity on a frequent basis (a minimum of ‘several times a week’) were invited to participate in the study. Individuals were excluded from participating if they were pregnant/breastfeeding, taking pain medication, experiencing serious and/or painful health conditions, had experience of a dental professional hygiene visit within the past 14 day (or periodontal surgery within the past 6 months), were suffering from xerostomia or if they had a recent history of substance misuse. Individuals who did not regularly access routine dental appointments were also excluded. The screening questionnaire therefore aimed to identify and exclude individuals whose sensitivity could have been caused by alternative factors/clinical pathology.

Participants who met the inclusion criteria were provided with additional study information. Upon obtaining informed consent, participants were asked to complete a text survey at 2 pm every day for 2 weeks. Participants also completed an online daily diary every evening.

The online diary was used to assess dentine hypersensitivity-related coping behaviours, oral health behaviours, perceived coping efficacy, pain predictions and mood on a daily basis.

Dentine hypersensitivity-related coping behaviours were assessed using the mean score from the 12 item ‘adaptation’ subscale from the revised Dentine Hypersensitivity Experience Questionnaire [11]. An example item is ‘Today, when I ate some foods I have made sure they didn’t touch certain teeth’ (1 = strongly disagree to 7 = strongly agree). The participant’s use of sensitivity toothpaste was assessed using the item: ‘Have you used sensitivity toothpaste designed for sensitive teeth today (or do you expect to have by the end of the day?’ (No = 1, Yes = 2).

Perceived efficacy of pain coping was assessed on a 5-point scale (1 = not at all, 2 = slightly, 3 = somewhat, 4 = moderately, 5 = very much) in response to the item ‘Considering all the things you did or thought today to contend with your pain, how much were you able to alleviate your pain by doing or thinking these things?’ [14]. Higher scores reflected greater perceived coping efficacy. Pain frequency prediction was assessed via the online diary by asking respondents ‘How often do you expect you will experience these sensations tomorrow?’

The Short Positive and Negative Affect Scale (PANAS) assessed daily mood [24] on two primary dimensions: positive (PANAS-P, 5 items) and negative affect (PANAS-N, 5 items). Items are rated on a 5-point scale (1 = very slightly or not at all to 5 = extremely). Total scores for each mood dimension range from 5 to 25. In the current study state mood was assessed by asking participants to rate how they had felt over the course of the day.

Pain frequency was recorded at two points throughout the day by enquiring ‘How many times have you experienced sensations up until 2 pm today?’ (text survey) and ‘How many times have you experienced sensations since 2 pm today?’ (online diary). At the end of each day the online survey assessed overall pain intensity, bothersomeness and tolerability for that day using three visual analogue scales (scored 1–10) (11). Functional limitations were assessed using mean scores from the 4 item ‘restrictions’ subscale of the DHEQ [11].

On completion of the study participants were asked to provide feedback on their experiences (see Table 1). Items included ‘Has taking part in the study changed your experience of sensitivity?’ and ‘How easy or difficult was it to complete the online daily diaries?’. Participants were paid a small financial incentive for each diary entry submitted.

**Table 1:** Participant methodology feedback

Feedback item within follow-up questionnaire	Number of people (*N* = 99)
Has taking part in the study changed your experience of sensitivity?	
Yes	43
Don’t know	20
No	36
How easy or difficult was it to complete the daily text survey?	
Very difficult	0
Difficult	1
Neither easy not difficult	1
Easy	11
Very easy	86
Did completing the text survey mid afternoon help you remember how many times you had experienced sensitive teeth that day?	
Yes	83
Don’t know	7
No	9
How easy or difficult was it to complete the onl	
Very difficult	0
Difficult	5
Neither easy not difficult	8
Easy	34
Very easy	52
If, on occasions, you completed the daily diary in the evening how easy or difficult was it to remember the experiences related to your sensitive teeth that day?	
Very difficult	0
Difficult	4
Neither easy not difficult	13
Easy	44
Very Easy	38
If, on occasions, you completed the daily diary following day but before 10 am how easy or difficult was it to remember the experiences related to your sensitive teeth the previous day?	
Very difficult	2
Difficult	10
Neither easy not difficult	15
Easy	37
Very Easy	15
Not applicable	20
If, on occasions, you completed the daily diary after 10 am the following day how easy or difficult was it to remember the experiences related to your sensitive teeth the previous day?	
Very difficult	5
Difficult	7
Neither easy not difficult	17
Easy	18
Very Easy	9
Not applicable	43
How did you feel about the duration of the study?	
I felt the duration of the study was about right	46
I felt two-weeks was too long a period of time to be completing diary entries each day	3
I would have been willing to complete more daily diaries over a longer period of time	50

### Models and analysis

Model 1 aimed to examine the relationship between coping, appraisal, pain experience and functional impacts associated with dentine hypersensitivity using lagged analysis (e.g. examining how coping and appraisal from day 1 influenced outcomes on day 2). Model 2 aimed to examine the relationship between mood, coping and pain experience using longitudinal data using a cross sectional method of analysis (mean scores across the 14 days were calculated for each variable). Based on sample size concerns a pre-selection criteria was employed for Model 2 and only significant baseline predictors of pain experience (*p* < 0.20) were entered into the model (pre-selection based on Spearman & Pearson correlations).

The statistical modelling procedure of Path Analysis was used (AMOS 18.0) to test the extent to which the models fitted the dataset. Bootstrapping was conducted and bias corrected 95 % confidence interval (CI) bootstrap percentiles were used to interpret the results. This approach is recommended for sample sizes of less than 20 [25, 26]. Due to the inclusion of a dichotomous variables (use of sensitivity toothpaste: 1 = no; 2 = yes) in both models, the ADF estimation method was used.

## Results

Two-hundred and eighty individuals expressed an interest in the study, of whom 101 respondents fulfilled the inclusion criteria. Most were female (*N* = 69) and ranged between 18 years and 63 years (mean age 26.3 years, SD = 8.6).

Scores for the daily variables are summarised in Table 2. Dentine hypersensitivity-related coping behaviours reported most frequently on day one included ‘today, when I ate some foods I have made sure they don’t touch certain teeth’ and ‘today, I had to change the way I drank or ate certain things’ (54 and 51 %, respectively). Just over a third of participants used sensitivity toothpaste but daily use varied over the 2-week daily diary study (range 34 to 43 %, average across 14 days = 37 %). When asked ‘considering all the things you did or thought today to contend with your pain how much were you able to alleviate pain by doing or thinking these things?’ 18 % reported ‘not at all’, 26 % reported ‘slightly’, 20 % reported ‘somewhat’, 16 % reported ‘moderately’ and 19 % reported ‘very much’ (on day one) revealing a wide variety of responses related to coping efficacy.

**Table 2:** Daily mean scores

Day	Positive mood mean (SD)	Negative mood mean (SD)	Coping mean (SD)	Efficacy mean (SD)	Pain frequency prediction mean (SD)	Pain frequency mean (SD)	Pain intensity mean (SD)	Pain bothersome-ness mean (SD)	Pain (in) tolerability mean (SD)	Functional limitations mean (SD)
1	15.1 (4.3)	8.4 (4.0)	2.8 (1.0)	2.9 (1.4)	2.8 (2.9)	3.0 (3.0)	3.9 (1.7)	3.4 (1.8)	2.7 (1.7)	2.7 (1.3)
2	14.6 (4.9)	8.3 (4.1)	2.8 (1.3)	2.9 (1.4)	2.5 (1.9)	2.9 (3.2)	3.5 (2.0)	3.3 (2.2)	3.0 (2.1)	2.8 (1.6)
3	14.4 (5.1)	8.3 (4.1)	2.8 (1.2)	2.9 (1.4)	2.3 (1.9)	2.7 (3.5)	3.4 (2.0)	3.1 (2.1)	2.9 (2.1)	2.8 (1.6)
4	14.0 (5.0)	8.8 (4.5)	2.8 (1.2)	3.0 (1.4)	2.3 (1.8)	2.6 (2.6)	3.3 (1.7)	3.0 (1.7)	2.7 (1.7)	2.8 (1.2)
5	13.8 (5.0)	7.4 (3.4)	2.9 (1.5)	3.0 (1.5)	2.3 (1.8)	2.8 (2.6)	3.6 (2.0)	3.3 (2.1)	3.1 (2.0)	3.0 (1.6)
6	14.0 (5.3)	7.1 (3.0)	2.9 (1.2)	3.0 (1.3)	2.3 (1.8)	2.9 (3.7)	3.3 (1.8)	3.1 (1.8)	2.9 (1.7)	3.0 (1.5)
7	14.0 (5.3)	7.8 (3.5)	2.9 (1.2)	3.0 (1.4)	2.3 (1.9)	2.7 (2.7)	3.3 (1.8)	3.0 (1.9)	2.7 (1.7)	3.1 (1.6)
8	14.3 (4.8)	8.0 (4.1)	3.0 (1.4)	3.0 (1.4)	2.5 (2.4)	3.2 (5.0)	3.5 (2.0)	3.1 (2.0)	2.9 (1.9)	3.1 (1.7)
9	14.1 (5.0)	8.0 (4.3)	3.0 (1.4)	2.8 (1.3)	2.4 (1.9)	2.6 (2.3)	3.3 (2.0)	3.3 (2.1)	3.0 (2.0)	3.1 (1.6)
10	14.2 (5.1)	7.7 (4.0)	3.0 (1.4)	2.8 (1.4)	2.3 (1.8)	2.4 (2.4)	3.3 (1.8)	3.0 (1.8)	2.9 (1.8)	2.9 (1.6)
11	15.0 (5.1)	7.4 (3.0)	3.0 (1.4)	3.1 (1.3)	2.4 (2.0)	3.0 (2.6)	3.2 (1.7)	3.0 (1.8)	3.0 (1.9)	2.9 (1.6)
12	15.0 (5.1)	6.9 (3.3)	2.9 (1.4)	2.9 (1.3)	2.2 (1.8)	2.5 (2.7)	3.1 (1.9)	2.9 (1.9)	2.7 (1.4)	2.7 (1.5)
13	13.1 (4.8)	7.3 (3.5)	3.1 (1.3)	2.9 (1.3)	2.4 (1.9)	2.7 (2.9)	3.1 (1.7)	2.8 (1.6)	2.7 (1.6)	2.9 (1.5)
14	13.4 (5.1)	7.7 (4.0)	3.1 (1.3)	2.8 (1.3)	2.4 (1.9)	3.1 (3.2)	3.3 (1.9)	2.9 (1.8)	2.7 (1.9)	3.0 (1.6)
14 day mean	14.2 (3.3)	7.8 (2.5)	2.9 (1.1)	2.9 (1.1)	2.4 (1.7)	2.8 (2.3)	3.3 (1.3)	3.1 (1.4)	2.8 (1.4)	2.9 (1.1)

**Fig. 1:**
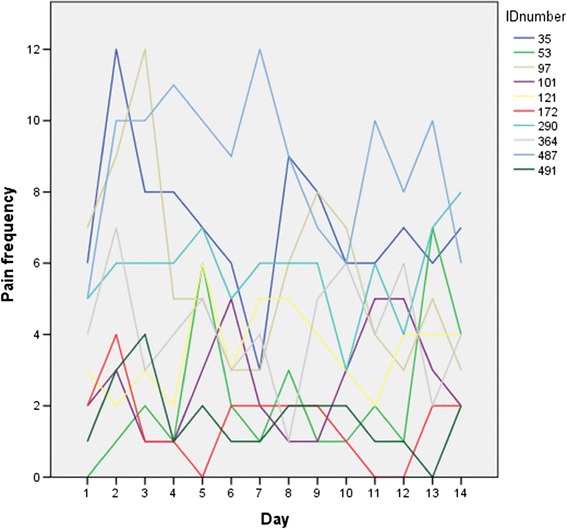
Random sample (10 %) of individuals’ pain frequency trajectories

The functional limitations reported most frequently on day one of the diary study was ‘today, having sensations in my teeth took a lot of pleasure out of eating and drinking’ (33 %) and the least frequent limitation reported was ‘today, there have been times when I couldn’t finish my meal because of the sensations’ (7 %). Mean pain frequency over the 2-week diary study was 2.8 (SD = 2.3) and the median pain frequency value ranged between 2 and 3 pain sensations per day. However, the number of pain sensations individuals reported ranged from 0–11 (day 1) to 0–43 (day 8) highlighting the between-person variability in dentine hypersensitivity pain over the 2-week period. Examination of individual trajectories also highlighted considerable within-person variation in pain frequency over the same period (Fig. 1).

### Do oral health-related coping, perceived coping efficacy and pain predictions successfully predict following day health outcomes in dentine hypersensitivity?

#### Model 1: coping strategies, appraisal and following day impacts

The model hypothesised 10 direct pathways based on previous findings from the stress and coping literature. To examine the relationship between oral health coping and outcomes for dentine hypersensitivity, pathways between dentine hypersensitivity-related coping behaviours and the following factors were examined: use of sensitivity toothpaste (path 1); perceived coping efficacy (path 2); pain predictions (path 3) and following day functional limitations and pain frequency (paths 4 & 5). Relationships between use of sensitivity toothpaste and pain predictions (path 6) and the primary outcome of pain were also examined (paths 6 & 7). To examine the role cognitive appraisals play in dentine hypersensitivity relationships between perceived coping efficacy and pain predictions (path 8) and between pain predictions and following day pain frequency (path 9) were examined. Finally, the relationship between pain frequency and functional limitations was also investigated to examine whether these health outcomes are inter-related in dentine hypersensitivity sufferers (path 10).

In order to examine whether pathways identified in Model 1 were ‘stable’ over the 2-week period, seven identical validation models were examined using ‘pairings’ of discrete daily diary data (e.g. day 1 to 2, days 3 to 4 etc.). Model 1 did not differ significantly from the data across the seven daily pairings (*p* > 0.05), suggesting that the relationships between the key variables remained stable over the study period. Goodness of fit indices were all acceptable (Table 3) and significant pathways for the different days of interest are presented in Table 4. Variables included within the models accounted for between 23 and 66 % of the variance in pain frequency and 30 & 54 % of the variance for functional limitations.

**Table 3:** Goodness of fit indices for Model 1 and Model 2

Model 1 (*N* = 101)	Goodness of fit indices					
	X^2^/df (*p* > .05)	CMIN/df (<2.0)	IFI (>0.95)	RMSEA (<0.08)	SRMR (<0.08)	Criteria fitted
Day 1–2	**3.93/5 (** ***p*** **= 0.56)**	**0.79**	**1.02**	**0.00**	**0.07**	5
Day 3–4	**2.11/5 (** ***p*** **= 0.83)**	**0.42**	**1.09**	**0.00**	**0.03**	5
Day5–6	**3.16/5 (** ***p*** **= 0.68)**	**0.63**	**1.04**	**0.00**	**0.03**	5
Day7–8	**4.57/5 (** ***p*** **= 0.47)**	**0.91**	**1.01**	**0.00**	**0.04**	5
Day9–10	**2.99/5 (** ***p*** **= 0.70)**	**0.60**	**1.02**	**0.00**	**0.03**	5
Day 11–12	**6.33/5 (** ***p*** **= 0.28)**	**1.26**	**0.99**	**0.05**	**0.06**	5
Day13–14	**4.09/5 (** ***p*** **= 0.54)**	**0.82**	**1.02**	**0.00**	**0.05**	5
**Model 2 (** ***N*** **= 101)**	**1.95/3 (** ***p*** **= 0.58)**	**0.65**	**1.00**	**0.00**	**0.03**	5

Note: Figures in bold are those which meet the model-fitting criteria

**Table 4:** Significant pathways between oral health coping, appraisal and following day impacts (Model 1)

Daily models (*N* = 101)	Significant pathways	Total β value (bootstrap bias corrected 95 % CI)	Indirect β value (bootstrap bias corrected 95 % CI)	Direct β value (bootstrap bias corrected 95 % CI)
Days 1–2	D1 coping^a^ → D1 perceived efficacy D1 coping → D1 pain prediction D1 coping → D2 pain frequency D1 coping → D2 functional D1 pain prediction → D2 pain frequency	.27** (.06 to .45) .27** (.07 to .70) .45** (.24 to .65) .53** (.32 to .67) .68** (.46 to .83)	-- n/s .18* (.05 to .84) .18* (.05 to .35) --	.27** (.06 to .45) .30* (.06 to .72) .27* (.03 to .41) .35* (.16 to .54) .68** (.46 to .83)
Days 3–4	D3 coping → D3 pain prediction D3 coping → D4 pain frequency D3 coping → D4 functional D3 pain prediction → D4 pain frequency D3 pain prediction → D4 functional D4 pain frequency → D4 functional	.31** (.10 to .50) .23* (.03 to .41) .32** (.08 to .50) .61** (.35 to .76) .28** (.11 to .44) .46** (.24 to .62)	-- .20** (.07 to .34) .10* (.02 to .20) -- .28** (.11 to .44) --	.31** (.10 to.50) n/s .22* (.02 to .38) .61** (.35 to .76) - .46** (.24 to .62)
Days 5–6	D5 coping → D5 pain prediction D5 coping → D6 pain frequency D5 coping → D6 functional D5 pain prediction → D6 pain frequency D5 pain predictions → D6 functional D6 pain frequency → D6 functional	.34** (.15 to .50) -- .38** (.17 to .54) .63* (.21 to .74) .35* (.23 to .43) .56** (.41 to .65)	n/s .22** (.06 to .35) n/s -- .35* (.23 to .43*) --	.33** (.13 to .50) n/s .31** (.14 to .46) .63** (.21 to .74) -- .56** (.41 to .65)
Days 7–8	D7 coping → D7 pain prediction D7 coping → D8 functional D7 coping → pain frequency D7 pain prediction → D8 pain frequency D7 pain predictions → D8 functional D8 pain frequency → D8 functional	.30** (.13 to .46) .45** (.24 to .60) -- .46* (.33 to .67) .16* (.06 to .23) .34** (.15 to .46)	-- -- .15* (.03 to .26) -- .16* (.06 to .23) --	.29** (.11 to .44) .42** (.25 to .56) n/s .46* (.33 to .67) -- .34** (.15 to .46)
Days 9–10	D9 coping → D9 pain prediction D9 coping → D10 pain frequency D9 coping → D10 functional D9 pain prediction → D10 pain frequency D9 pain predictions → D10 functional D10 pain frequency → D10 functional	.37** (.18 to .53) .26** (.07 to .42) .40** (.40 to .71) .69* (.40 to .80) .17** (.05 to .28) .25** (.09 to .41)	n/s .24** (.09 to .38) .06** (.02 to .15) -- .17** (.05 to .28) --	.34** (.14 to 51) n/s .52** (.33 to .66) .69* (.40 to .80) -- .25** (.09 to .41)
Days 11–12	D11 coping → D11 pain prediction D11 coping → D12 pain frequency D11 coping → D12 functional D11 use of toothpaste → D12 pain frequency D11 use of toothpaste → D12 functional D11 pain prediction → D12 pain frequency D12 pain frequency → D12 functional	.41** (.22 to .56) .36** (.18 to .53) .68** (.42 to .82) .29** (.09 to .47) .09* (.02 to .20) .62* (.41 to .80) .31* (.01 to .50)	n/s .31** (.18 to .44) .11* (.02 to .19) n/s .09* (.02 to .20) n/s n/s	.35** (.18 to .51) n/s .57** (.31 to .77) .19* (.02 to .37) -- .64* (.41 to .80) .31* (.01 to .50)
Days 13–14	D13 coping → D13 pain prediction D13 coping → D14 pain frequency D13 coping → D14 functional D13 pain prediction → D14 pain frequency	.41** (.24 to .57) .40** (.22 to .61) .61** (.40 to .76) .69** (.56 to .83)	n/s .28** (.17 to .44) .14* (.00 to .27) --	.37** (.18 to .55) n/s .47** (.24 to .72) .69** (.56 to .83)

^a^dentine hypersensitivity-related coping behaviours

*p < 0.05, **p < 0.01

**Fig. 2:**
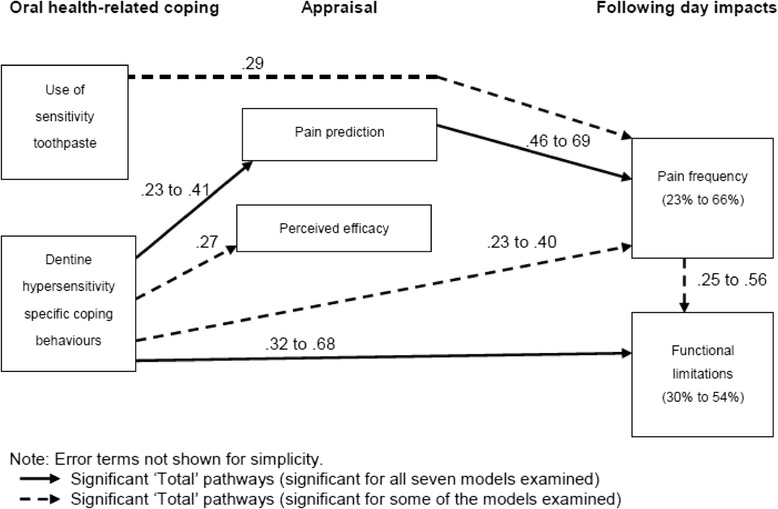
Significant total pathways between oral health coping behaviours, appraisal and following day impacts proposed within Model 1

Three significant direct pathways and one indirect pathway remained significant across all daily pairings (Table 4). Individuals using more dentine sensitivity-related coping behaviours were more likely to predict that they would experience more painful sensations the following day and were more likely to experience more functional limitations the following day. The prediction of more sensations was also associated with more pain the following day. Significant total effects, which existed when combining both direct and indirect pathways within the model can be seen in Fig. 2.

### Are daily mood and oral health behaviours associated with dentine hypersensitivity-related pain experience?

#### Model 2: mood, coping and pain experience

Positive mood was unrelated to the other variables so was not entered into the Path Analysis. The nine direct pathways hypothesised within the model were based on previous findings from the stress and coping literature. To examine the relationship between mood, oral health-related coping and oral health experiences five direct pathways between negative mood and use of sensitive toothpaste (path 1), pain frequency (path 2), pain intensity (path 3), pain tolerability (path 4) and pain bothersomeness (path 5) were examined. The relationship between the use of sensitivity toothpaste and pain was also examined (path 6). Finally, to examine how pain experiences of dentine hypersensitivity are inter-related, relationships between pain frequency and pain intensity (pain 7), bothersomeness (path 8) and tolerability (path 9) were all examined.

Model 2 did not differ significantly from the observed data (x2 = 1.95, df = 3, *p* = 0.58) and the goodness of fit indices were all excellent (Table 3). The variables included within the model accounted for 12 % of variance in pain frequency, 21 % of the variance for pain intensity, 13 % of the variance for pain tolerability and 18 % of the variance in pain bothersomeness.

**Fig. 3:**
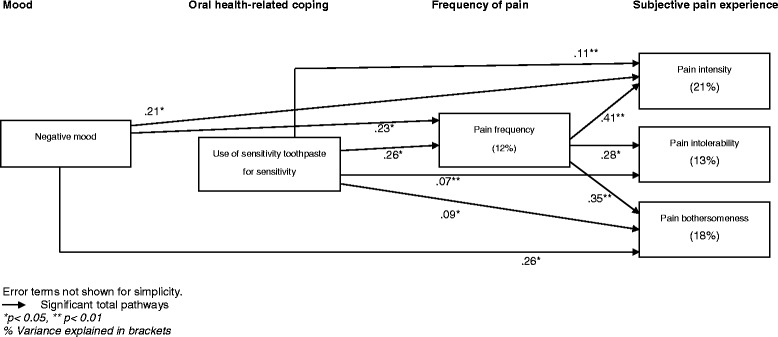
Significant total pathways between mood, oral health coping behaviours and pain experience in individuals with dentine hypersensitivity (Model 2)

The model contained five significant direct pathways and six indirect pathways (Table 5). More negative mood throughout the 2-week period was associated with more frequent painful sensations. Frequent sensations were associated with more intense pain, bothersomeness and less tolerability. Individuals who typically used sensitivity toothpaste reported more frequent pain. Significant total effects, which existed when combining both direct and indirect pathways can be seen in Fig. 3.

**Table 5:** Significant direct, indirect and total pathways between mood, toothpaste use and pain experience proposed within Model 2

Significant pathways	Direct pathways		Indirect pathways		Total pathways	
	β value	Bootstrap bias corrected 95 % CI	β value	Bootstrap bias corrected 95 % CI	β value	Bootstrap bias corrected 95 % CI
Negative mood → Use of sensitivity toothpaste Negative mood → Pain frequency Negative mood → Pain intensity Negative mood → Pain intolerability Negative mood → Pain bothersomeness	-.08 .24* .12 .17 .18	-.29 to .18 .05 to .42 -.06 to .37 -.07 to .45 -.00 to .42	-- -.02 .09* .06* .08*	-- -.09 to .05 .02 to .20 .01 to .16 .01 to .17	-.08 .23* .21* .23 .26*	-.29 to .18 .02 to .43 .02 to .54 -.02 to .51 .06 to .50
Use of sensitivity toothpaste → Pain frequency Use of sensitivity toothpaste → Pain intensity Use of sensitivity toothpaste → Pain intolerability Use of sensitivity toothpaste → Pain bothersomeness	.26* -- -- --	.04 to .47 -- -- --	-- .11** .07** .09**	-- .02 to .25 .01 to .18 .02 to .20	.26* .11** .07** .09**	.04 to .47 .02 to .25 .01 to .18 .02 to .20
Pain frequency → Pain intensity Pain frequency → Pain intolerability Pain frequency → Pain bothersomeness	.41** .28* .35**	.22 to .58 .07 to .45 .18 to .51	-- -- --	-- -- --	.41** .28* .35**	22 to .58 .07 to .45 .18 to .51

*p < 0.05, **p < 0.01

### Participants’ experiences of using daily diary methodology to examine dentine hypersensitivity

The number of participants who completed daily diaries on each of the study days ranged from 98 to 101. Almost half of participants felt that taking part in diary study had changed their experience of sensitivity, largely because it changed their awareness of how frequently they experienced their sensations:

*“It has made me realise that I do not feel sensations as often as I thought”*

*“I think I notice any sensations in my teeth more, and am probably generally more aware of my oral health as a result”*

Some perceptions and understanding of the oral condition had changed since participating in the study (e.g. understanding the triggers of dentine sensitivity):

*“I've begun to notice that it varies with my mood, I'm not sure how much one influences the other!”*

For some individuals, participating had helped them develop ways of coping with the pain or accepting their condition:

*“Made me think about the frequency and methods of dealing with it”*

Most participants felt that receiving the text survey mid-afternoon helped them recall information on their pain experiences and found the daily diary study easy to complete. However, they found it more difficult to complete the following day.

## Discussion

This research revealed how psychological factors may play an important role in the day-to-day oral experiences of people with dentine hypersensitivity. These factors, coping strategies and experiences, fluctuated within individuals throughout the study period and thus the daily diary approach allowed sufficient precision to detect associations between these fluctuations.

Previous diary research has found mixed results in relation to the use of coping and subsequent pain levels. For example, the use of cognitive reframing strategies in the morning predicted less evening pain experience, whereas morning use of active problem solving predicted more evening pain [27]. Within the current study, the use of dentine hypersensitivity-related coping behaviours predicted worse pain and functional limitations the following day. However, there was some evidence that greater use of specific coping adaptations were positively associated with higher levels of perceived coping efficacy. Therefore, an explanation for the relationship between coping and following day pain experience could be that those individuals who felt the need to use dentine sensitivity related-coping behaviours were already experiencing more pain and found the coping adaptations did help them control this pain. This highlights the importance of examining individuals’ coping appraisals as a mechanism for aiding our understanding into the complex, and probable bi-directional, nature of the relationship between coping and pain.

Pain predictions were strongly associated with subsequent pain experience, indicating that despite its fluctuating nature, pain is predictable in dentine hypersensitivity. The mechanisms through which pain predictions influence subsequent pain experience warrants further examination [28]. Individuals with worse negative daily mood were also more likely to report more pain, of a higher intensity, less tolerability and greater bothersomeness. This is the first research to highlight the role of mood in individuals’ experiences of this specific type of dental pain. These findings are compatible with previous research which has found similar effects in relation to chronic pain [17, 18]. Whilst the causality of the relationship between mood and pain is subject to debate, state negative affect can increase symptom perception and reporting [29, 30].

The explained variance in pain frequency and functional limitations differed considerably across the 2-week period (23–66 % and 30–54 %,respectively). This variation in the explanatory power of coping and appraisal factors on dentine hypersensitivity experiences provides evidence that the factors and processes that influence these daily oral health experiences may also fluctuate on a daily basis. It is also important to recognise that there were only small to moderate associations between pain frequency and the other subjective elements of pain experience reported (intensity, bothersomeness and intolerability). This suggests that only measuring the frequency with which someone experiences pain may fail to provide a reliable and holistic account of the individual’s pain experience.

A major strength of the current study was its prospective design of the research with frequent data collection, which is less likely to be subject to recall error or bias. This success was reflected in the high response rate throughout the daily data collection and the positive feedback from participants regarding different aspects of the study’s design which is consistent with findings from studies using similar methods [31, 32]. However, participants’ response times became increasingly delayed during the study, supporting previous research describing the cumulative burden of completing daily diaries and decreased reliability of responses over time [31]. The data revealed that most individuals felt the text surveys had acted as a useful aid to their pain recall throughout the day, highlighting the usefulness of signal-contingent methods in daily diaries [33]. Incentives were used in the current study and this could have also contributed to the high response rate. The use of incentives are associated with higher levels of engagement levels in research studies which employ diary methods [34].

The potential value of utilising e-diaries (e.g. via a phone app) as a clinical assessment tool could also be examined in future research. Prospective diary methods could be used to collect accurate data on patient’s dentine hypersensitivity-related pain and coping, this information could aid the patient’s and clinician’s understanding of the patient’s own unique experience of the condition and the possible factors that may help them effectively manage their symptoms. However, further evaluation of the feasibility and utility of using this type of assessment tool in a clinical setting is required.

Whilst these data start to explain the experience of dentine hypersensitivity, the research was not without limitations. Individuals were university students and staff and may not represent the general population and this could affect the generalisability of the findings. For example, the high response rate and level of engagement with the daily diaries may not be replicated in studies that use different groups of the population. The methods employed (e.g. internet diaries and text surveys) also excluded individuals who did not have daily access to the internet and a mobile phone, which could have further biased the sample.

It is important to recognise that participants self-diagnosed their dentine hypersensitivity and no clinical examinations were undertaken to identify alternative clinical pathologies which could have been responsible for pain experiences. Oral conditions, which have symptoms similar to dental hypersensitivity, should be excluded to ensure an accurate diagnosis of dentine hypersensitivity can be made [23]. Within the current study it is possible that some individuals could have been experiencing oral pain caused by a different pathology. However, a pre-study screening questionnaire and strict exclusion criteria based on Holland et al.’s guidelines [23] were employed. Irregular dental attendance was one of the exclusion criteria employed to minimise the likelihood that individuals with untreated dental disease/conditions (e.g. cavities or trauma) could participate in the study. It is also possible that participants self-monitoring effects could have influenced the data obtained. Self-monitoring is an intervention in its own right and several psychological models highlight the impact selective attention to bodily symptoms can have on the experiencing of symptoms [35, 36]. Therefore, the issues associated with self-monitoring should be recognised by researchers and clinicians who employ daily diary methods.

## Conclusions

These data offer unique insights into the experiences of people with dentine hypersensitivity. The study revealed how dentine hypersensitivity experiences fluctuate on a daily basis, which has significant implications for the how these oral health experiences can be measured in a meaningful and reliable way. The use of prospective methods of data collection, such as daily diaries, can develop understanding of the day-to-day impacts caused by oral conditions and the complex relationships that exist between transient states and experiences such as psychological factors and dental pain. This information can place clinicians in a stronger position to manage their patients’ oral health conditions.

## Abbreviations

ADF: Asymptotically distribution-free
DHEQ: Dentine hypersensitivity experience questionnaire
PANAS: Positive and negative affect scale
